# ASO Author Reflections: Tumor Burden and the Prognostic Relevance of Adequate Lymph Node Dissection in Intrahepatic Cholangiocarcinoma

**DOI:** 10.1245/s10434-026-20058-9

**Published:** 2026-06-23

**Authors:** Jun Kawashima, Yuki Homma, Itaru Endo, Timothy M. Pawlik

**Affiliations:** 1 Department of Gastroenterological Surgery, Yokohama City University School of Medicine, Yokohama, Japan; 2 Department of Surgery, The Urban Meyer III and Shelley Meyer Chair for Cancer Research, The Ohio State University Wexner Medical Center and James Comprehensive Cancer Center, Columbus, OH USA

## Past

Among patients with intrahepatic cholangiocarcinoma (iCCA), lymph node metastasis has been associated with poor outcomes even after curative-intent resection.^1^ Accordingly, several guidelines recommend regional lymph node dissection (LND) as part of the surgical procedure.^2^ Although LND is well established for postoperative prognostic assessment, its therapeutic benefit remains controversial. Theoretically, LND may contribute not only to accurate nodal staging, but also to clearance of regional nodal disease. Therefore, the clinical relevance of LND may be greater among patients with more aggressive tumor biology. Because morphologic tumor burden may serve as a surrogate marker of underlying tumor biology and aggressiveness,^3^ we hypothesized that tumor burden score, a composite index incorporating maximum tumor size and tumor number, may modify the prognostic impact of regional LND for iCCA.

## Current

In our international multi-institutional retrospective study, 1,558 patients undergoing curative-intent resection for iCCA were analyzed.^4^ Overall, 872 patients underwent LND, whereas only 322 patients underwent adequate LND, defined as retrieval of at least six lymph nodes. On multivariable Cox regression analysis, a significant interaction was observed between tumor burden score (TBS) and adequate LND, indicating that the prognostic relevance of adequate LND varied according to tumor burden. Among patients with low TBS (<5.0), adjusted overall survival did not differ according to adequate LND status. In contrast, among patients with high TBS (≥5.0), adequate LND was associated with improved adjusted overall survival. Similar findings were observed for recurrence-free survival. These data suggest that guideline-concordant nodal evaluation may have particular oncologic relevance among patients with greater tumor burden.

## Future

Adequate LND for iCCA remains inconsistently achieved in real-world practice. Indeed, recent studies have demonstrated that only 10–25% of patients undergoing curative-intent resection for iCCA achieve guideline-concordant nodal evaluation.^5^ The current findings suggest that inadequate nodal evaluation may be particularly consequential among patients with high TBS, who may otherwise derive oncologic benefit from adequate LND. Future efforts should aim to improve adherence to guideline-concordant LND, especially in patients with high TBS, while prospectively validating the present findings. Ultimately, this approach may help inform surgical strategies tailored to tumor morphology and underlying tumor biology.

## References

[1] Kawashima J, Akabane M, Woldesenbet S, et al. Oncological resectability criteria for intrahepatic cholangiocarcinoma: a preoperative framework for multidisciplinary management. *Ann Surg Oncol*. 2025;32(10):7141–51. 10.1245/s10434-025-17776-x. 40634809 10.1245/s10434-025-17776-x PMC12454504

[2] European Association for the Study of the Liver. EASL-ILCA Clinical Practice Guidelines on the management of intrahepatic cholangiocarcinoma. *J Hepatol*. 2023;79(1):181-208. 10.1016/j.jhep.2023.03.010 10.1016/j.jhep.2023.03.010 37084797

[3] Tsilimigras DI, Hyer JM, Paredes AZ, et al. Tumor burden dictates prognosis among patients undergoing resection of intrahepatic cholangiocarcinoma: a tool to guide post-resection adjuvant chemotherapy? *Ann Surg Oncol*. 2021;28(4):1970–8. 10.1245/s10434-020-09393-7. 33259043 10.1245/s10434-020-09393-7

[4] Kawashima J, Sahara K, Yuza K, et al. Tumor burden modifies the prognostic impact of adequate lymph node dissection in patients undergoing curative-intent resection for intrahepatic cholangiocarcinoma. *Ann Surg Oncol*. 2026. 10.1245/s10434-026-19995-2. 42289631 10.1245/s10434-026-19995-2 PMC13452779

[5] Moazzam Z, Alaimo L, Endo Y, Lima HA, Pawlik TM. Predictors, patterns, and impact of adequate lymphadenectomy in intrahepatic cholangiocarcinoma. *Ann Surg Oncol*. 2023;30(4):1966–77. 10.1245/s10434-022-13044-4. 36622527 10.1245/s10434-022-13044-4

